# Case Report: Zilucoplan in refractory myasthenic crisis and its prodromal stages: a novel therapeutic perspective

**DOI:** 10.3389/fnins.2026.1886744

**Published:** 2026-07-29

**Authors:** Liliana Bevilacqua, Carmen Erra, Dario Ricciardi, Francesco Tuccillo, Francesco Habetswallner, Maria Teresa Pellecchia, Paolo Barone, Claudia Vinciguerra

**Affiliations:** 1 Department of Medicine, Surgery and Dentistry “Scuola Medica Salernitana”, University Hospital “San Giovanni di Dio e Ruggi d’Aragona”, University of Salerno, Salerno, Italy; 2 Clinical Neurophysiology Unit, Azienda Ospedaliera di Rilievo Nazionale-Cardarelli, Naples, Italy; 3 IRCCS SYNLAB-SDN, Napoli, Italy

**Keywords:** complement inhibition, impending crisis, myasthenia gravis, myasthenic crisis, Zilucoplan

## Abstract

Myasthenic crisis (MC) represents life-threatening respiratory failure in myasthenia gravis (MG), while impending myasthenic crisis (iMC) denotes rapidly worsening weakness threatening ventilation however the condition is reversible with timely intervention. Complement inhibition has emerged as a targeted approach for acetylcholine receptor (AChR) antibody-positive MG. To date, evidence for Zilucoplan, a subcutaneous macrocyclic peptide C5 inhibitor, in acute settings remains limited. This study describes two Italian AChR-positive-MG patients experiencing severe, treatment-refractory exacerbations. One patient presented with overt MC requiring non-invasive ventilation, and the second with iMC and rapidly worsening bulbar dysfunction. Both patients received Zilucoplan 32.4 mg/day subcutaneously following failure of corticosteroids, intravenous immunoglobulin, and, in one case, multiple biologic agents. Zilucoplan provided rapid, robust, and durable improvement in refractory AChR-positive MG during both MC and iMC. Both patients maintained sustained remission with no adverse events under ongoing Zilucoplan therapy. Continuous complement blockade and easy-to-use subcutaneous administration may offer significant advantages for acute rescue and maintenance therapy. Early initiation could prevent progression from iMC to established MC. Larger prospective studies are warranted to define optimal use and timing of Zilucoplan in MG crisis management.

## Introduction

Myasthenic crisis (MC) represents the most severe manifestation of myasthenia gravis (MG), defined by respiratory failure requiring mechanical ventilation or severe bulbar dysfunction with high risk of aspiration (Gilhus et al., 2019; Roper et al., 2017). A related but less severe condition—impending myasthenic crisis (iMC)—refers to rapidly worsening weakness, particularly of bulbar or respiratory muscles that can lead to respiratory failure but in which ventilation can still be avoided with timely intervention (Sanders et al., 2016). Together, these states, account for significant morbidity and mortality, affecting approximately 15–20% of MG patients during their disease course (Gilhus et al., 2019; Roper et al., 2017).

Standard management of MC includes optimization of cholinesterase inhibitors, corticosteroids, and the use of fast-acting rescue therapies such as intravenous immunoglobulin (IVIg), or plasma exchange (PLEX). Despite these options, a subset of patients remains refractory, often requiring prolonged intensive care (Roper et al., 2017).

Complement inhibition has emerged as a targeted therapy in acetylcholine receptor (AChR) antibody-positive MG (San and Jacob, 2023). By preventing cleavage of complement component C5 and formation of the membrane attack complex (MAC), complement inhibitors protect the postsynaptic membrane and improve neuromuscular transmission. Zilucoplan, a subcutaneously self-administered macrocyclic peptide that binds C5, demonstrated rapid and sustained benefit in phase III trials (RAISE, RAISE-XT) with a good safety profile and easy self-administration (Howard et al., 2023; Howard et al., 2024).

However, the real-world evidence in crisis settings remains limited. Only a few reports describe rapid respiratory and bulbar recovery following Zilucoplan in refractory crises (Maruani et al., 2025; Ito et al., 2025). Against this background the study presents two additional AChR-positive cases—one with a defined MC and the other with an iMC—both unresponsive to conventional therapy and showing prompt clinical recovery after Zilucoplan initiation.

## Case 1

A 38-year-old Italian man with AChR-positive MG, diagnosed in 2013 and associated with a type B2 thymoma, was admitted to the Neurology Unit of Salerno Hospital in August 2025 for acute worsening of dysphagia, dyspnea, and generalized muscle weakness. His previous treatments included thymectomy, radiation therapy, corticosteroids, IVIg, plasma exchange (PLEX), and several monoclonal antibodies: Rituximab for 8 years, then Eculizumab and Ravulizumab for 2 years, and Efgartigimod (2 cycles administered 4 weeks apart), all providing only partial benefit with wearing-off phenomenon. The patient had completed the recommended meningococcal vaccination schedule before starting anti-complement therapy, and received booster doses according to current guidelines (Girmenia et al., 2023).

On admission, neurological examination revealed neck and proximal limb weakness, nasal speech, dysphagia, and dyspnea at rest with hypoxia. The bulbar and respiratory involvement was so severe to require the use of a nasogastric (NG) feed and non-invasive ventilation (NIV): a clinical picture consistent with overt MC. Clinical scores showed a Myasthenia Gravis Foundation America (MGFA) class V, with a Quantitative Myasthenia Gravis (QMG) score of 23/39 and a Myasthenia Gravis Activities of Daily Living (MG-ADL) score of 16/24.

Initially, IVIg treatment was started at a dosage of 0.4 g/kg/day for 5 days and prednisone 30 mg/day. However, his condition became complicated by the development of Takotsubo-like cardiomyopathy (left ventricular ejection fraction, LVEF 30%), requiring transfer to the cardiac intensive care unit. After 2 weeks following stabilization and recovery of cardiac function (LVEF 60%), he was readmitted to the Neurology because of persistent myasthenic symptoms and the onset of a concomitant bacterial pneumonia, confirmed by a CT scan performed in order to exclude any thymoma relapse, and appropriate antibiotic therapy was started. On day 30, a second course of IVIg (2 g/kg in 5 days) was attempted, showing no clinical improvement.

Given the patient’s refractoriness to previous therapies, subcutaneous Zilucoplan at dosage of 32.4 mg/day was initiated on the 45th day of hospitalization. The patient immediately experienced marked clinical improvement: respiratory function normalized, allowing non-invasive ventilation to be reduced to nocturnal use only after 4 days; dysarthria and dysphagia rapidly resolved, with NG tube removal and full oral feeding restoration after 5 days. A chest CT performed on the 52nd day from admission, that confirmed complete resolution of pneumonia.

**Figure 1:**
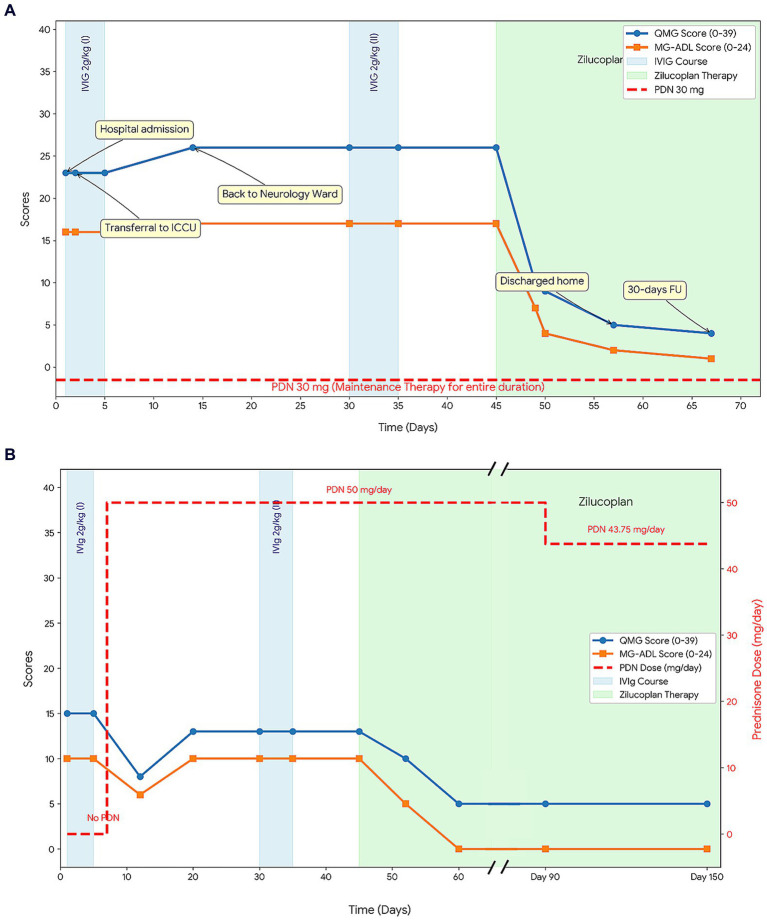
Clinical course of two MG patients with acetylcholine receptor-positive myasthenia gravis treated with Zilucoplan. The *x*-axis shows the patient’s days of hospitalization, while the *y*-axis reports the scores on the clinical scales. **(A)** A 38-year-old man with thymoma-associated MG during myasthenic crisis (MC). **(B)** An 81-year-old man with late-onset MG during impending myasthenic crisis (iMC). Green bars indicate intravenous immunoglobulin (IVIg) and the blue bar corticosteroid therapy. Red square indicates the clinical worsening or crisis onset, and green denotes Zilucoplan initiation, followed by rapid improvement—within 1 week in **(A)** and full bulbar recovery within 10 days in **(B)**. QMG, quantitative myasthenia gravis score; MG-ADL, myasthenia gravis activities of daily living scale; ICCU, intensive coronary care unit; IVIg, intravenous immunoglobulins; PDN, prednisone.

The patient was discharged on the 57th day, breathing spontaneously with only mild residual weakness (QMG 5/39, MG-ADL 2/24). At 30-days follow-up, the patient demonstrated near-complete recovery (QMG 4/39, MG-ADL 1/24). No adverse events were reported during ongoing Zilucoplan therapy (see Figure 1A).

## Case 2

An 81-year-old man with late-onset AChR-positive MG presented in July 2025 with rapidly progressive severe dysphagia and hypophonia consistent with iMC (MGFA class IVB) and was admitted to Neurology Unit of Cardarelli Hospital in Naples. His baseline clinical scores were QMG 15/39 and MG-ADL 10/24. Comorbidities included hypertension, benign prostatic hyperplasia, and previous ocular carcinoma.

The patient received 5 days of IVIg (0.4 g/kg/day) with transient improvement but relapsed after 20 days from admission, with worsening bulbar symptoms and shortness of breath. On neurological evaluation, he showed severe dysphagia requiring NG feeding, dysphonia, dyspnea at rest, weak cough without fatigable limb weakness (QMG 13/39, MG-ADL 10/24).

At 30 days after admission a second IVIg course (2 g/kg in 5 days) and prednisone 50 mg/day failed to improve symptoms. Because the patient declined hospital admission, subcutaneous Zilucoplan 32.4 mg/day was therefore started on day 45 as a more suitable therapeutic approach in the outpatient setting. Antibiotic prophylaxis for meningococcal infection was started with Rifampicin 600 mg/day.

After 1 week nasal speech resolved and he resumed soft food. By the second week, QMG was 5/39 and MG-ADL was 0/24. One-month follow-up confirmed full bulbar recovery and stable respiratory and limb strength (QMG 5/39, MG-ADL 0/24, prednisone tapered at dosage of 43.75 mg/day). He continued Zilucoplan therapy followed by meningococcal vaccination.

After 2 months, the patient continued to exhibit clinical stability (QMG 5/39, MG-ADL 0/24) under a gradual tapering regimen of prednisone, with the latest dose at 30 mg/day (see Figure 1B).

## Discussion

These cases highlight the potential role of Zilucoplan as a rescue therapy in severe and refractory AChR-positive MG, including both established MC and its prodromal stages. In both patients, initiation of Zilucoplan led to a rapid and sustained improvement in bulbar and respiratory function within 1 week, despite prior failure of corticosteroids, IVIg, and—in one patient—multiple advanced targeted therapies. They both reached minimal symptom expression (MSE) and maintained it throughout the whole follow-up. Such findings are consistent with emerging RWE data, that suggest Zilucoplan is well-tolerated and effective in most patients with AChR-positive gMG (Di Stefano et al., 2025).

Although conventional rescue treatments remain first-line, their often delayed or incomplete efficacy can prolong ventilation or lead to tracheostomy in refractory cases. In this context, the rapid pharmacodynamic effect of complement inhibitors suggests a potential role in acute care, even though current evidence is still limited to isolated reports and small case series (see Table 1) (Vinciguerra et al., 2023; Erra et al., 2025).

**Table 1:** Summary of reported cases of Zilucoplan use in myasthenic or impending crisis, including demographics, MG subtype, concomitant treatments, timing of intervention, and clinical outcomes.

Authors	Sex, Age	MG type	Clinical severity	Concomitant treatments	Timing of intervention	Scales at baseline	Scales at latest follow-up
Vinciguerra et al. (2023)	M, 38	TAMG	MC	2 courses of IVIg, PDN 30 mg	45 Days	MGFAV, QMG 21, MG-ADL 15	MGFA IIa, QMG 4, MG-ADL 1
Vinciguerra et al. (2023)	M, 81	LOMG	iMC	2 courses of IVIg, PDN 50 mg	45 Days	MGFA IVb, QMG 13, MG-ADL 10	MGFA IIb, QMG 5, MG-ADL 0
Maruani et al. (2025)	M, 81	LOMG	MC	3 courses of IVIg, MMF 2 g/d, PLEX, PDN 1 g/kg, RTX 1 g twice	7 Weeks	MGFA V	MGFA I, MG-ADL 0
Maruani et al. (2025)	M, 68	LOMG	MC	AZA 100 mg, IVIg, PDN 0.75 mg/kg, 18 PLEX sessions, RTX 1 g twice, MMF	2 Months	MGFA V	MGFA I, MG-ADL 0
Ito et al. (2025)	F, 83	LOMG	MC	Tacrolimus 2 mg/d, IVIg, IVMP 250–500 mg/d	11 Days	MGFA V, QMG 27, MG-ADL 23	MGFA IIIa, QMG 12, MG-ADL 7

TAMG, thymoma-associated myasthenia gravis; LOMG, late-onset myasthenia gravis; MC, myasthenic crisis; iMC, impending myasthenic crisis; MGFA, Myasthenia Gravis Foundation of America Classification; QMG, Quantitative Myasthenia Gravis score; MG-ADL, Myasthenia Gravis – Activities of Daily Living scale; IVIg, intravenous immunoglobulins; PDN, prednisone; MMF, mycophenolate mofetil; AZA, azathioprine; PLEX, plasma exchange; IVMP, intravenous methylprednisolone.

Zilucoplan acts inhibiting C5 cleavage and preventing MAC formation, thereby preserving post-synaptic integrity and rapidly restoring neuromuscular transmission (San and Jacob, 2023; Tang et al., 2023). Daily subcutaneous dosing ensures stable complement blockade, supporting early functional recovery. The rapid clinical improvement observed in both patients aligns with trial data showing early onset of benefit (Howard et al., 2023; Howard et al., 2024). Moreover, as a macrocyclic peptide, Zilucoplan can be administered concomitantly with other acute-phase therapies, such as IVIg and PLEX, maintaining complete complement inhibition without interference or the need for additional dosing (Howard et al., 2024).

Of note, the report includes two patients with distinct subtypes of AChR-positive MG: a young patient with thymoma-associated disease and an elderly patient with very-late-onset MG. Thymoma-associated MG is typically linked to a breakdown of central tolerance driven by the tumor microenvironment and a broader autoimmune milieu, whereas very-late-onset MG is thought to arise in a different context, characterized by immunosenescence and age-related loss of tolerance in the setting of an involuted thymus, often affecting frail patients with multiple comorbidities. The similarly rapid and remarkable clinical improvement observed after Zilucoplan treatment in both patients further supports the key role of terminal complement activation and the efficacy of complement inhibitors across different AChR-positive MG phenotypes.

In the first patient, the response to Zilucoplan was particularly remarkable, as it occurred after failure of two intravenous C5 inhibitors, which had provided only a partial response and showed a wearing-off effect. Indeed, there is recent evidence of clinically meaningful improvement on MG-ADL and QMG scores switching from intravenous C5-inhibitors, particularly Ravulizumab, to subcutaneous Zilucoplan (Freimer et al., 2025). The different ligand site and daily administration may enable a deeper and more stable complement suppression, thereby allowing symptom improvement in patients with unsatisfactory response to intravenous C5 inhibitors.

In the second patient, early initiation during iMC appeared to prevent progression to overt respiratory failure, underscoring the importance of timely recognition of crisis prodromes and prompt therapeutic intervention before clinical deterioration occurs. This aspect is particularly relevant in patients with very-late-onset MG, a vulnerable subgroup characterized by greater frailty, a higher burden of comorbidities, and limited functional reserve. In this subset of patients, prolonged hospitalization and extended invasive ventilation are associated with poorer functional recovery and lower rates of minimal symptom expression (MSE) achievement 1 year after a MC (Huan et al., 2026). Furthermore, the use of conventional rescue therapies is often constrained by comorbidities and carries an increased risk of treatment-related complications. In this context, the rapid and selective complement inhibition provided by Zilucoplan may be particularly advantageous as an early rescue strategy in this population.

Treatment was well tolerated, with no infectious, hematologic, or systemic adverse events, even in patients with high burden of comorbidity, pointing out its suitability when started in the ICU setting and in late-onset myasthenia gravis patients. Subcutaneous administration also allows seamless transition from acute rescue to outpatient maintenance therapy, which may be particularly advantageous in elderly or comorbid patients. Possible confounding factors should be acknowledged. Although Zilucoplan was initiated at least 15 days after the last IVIg course in both cases, a delayed contribution of IVIg or corticosteroids to the observed clinical improvement cannot be entirely excluded; such an effect may have been subsequently consolidated and sustained by Zilucoplan.

## Conclusion

Zilucoplan induced rapid, marked, and sustained improvement in two patients with severe AChR-positive MG—one with established crisis and one with iMC—both refractory to standard rescue therapies. Its rapid onset of action, consistent complement inhibition, and convenient subcutaneous administration make it a promising option for acute rescue and ongoing management in refractory MG that encompasses different patient profiles with various immunological backgrounds and a broad spectrum of comorbidities.

### Implication for clinical care

The early complement inhibition may prevent progression from iMC to a definite respiratory failure and support faster recovery, potentially reducing the need for invasive ventilation. Further prospective and multicenter studies are required to define the optimal timing, duration, and integration of Zilucoplan into the therapeutic algorithm for MC and its prodromal stages.

## Data Availability

The original contributions presented in the study are included in the article/supplementary material, further inquiries can be directed to the corresponding author.

## References

[ref1] Di Stefano V. Rini N. Antozzi C. Alboini P. E. Alonge P. Bevilacqua L. . (2025). Early real-life experience on Zilucoplan for generalized myasthenia gravis: ZILU25 multicenter observational study. J. Neurol. Sci. 479:125671. doi: 10.1016/j.jns.2025.125671, 41314023

[ref2] Erra C. Ricciardi D. Vinciguerra C. Fasolino A. Andreone V. Habetswallner F. . (2025). Eculizumab as treatment in refractory impeding and myasthenic crisis: a case series. Neurocrit. Care. 43, 685–690. doi: 10.1007/s12028-025-02272-7, 40295418

[ref3] Freimer M. Desai U. Govindarajan R. Kang M. K. Khan S. Khatri B. . (2025). Switching to subcutaneous Zilucoplan from intravenous complement component 5 inhibitors in generalised myasthenia gravis: a phase IIIb, open-label study. Ther. Adv. Neurol. Disord. 18:17562864251347283. doi: 10.1177/17562864251347283, 40620733 PMC12228924

[ref4] Gilhus N. E. Tzartos S. Evoli A. Palace J. Burns T. M. Verschuuren J. J. G. M. (2019). Myasthenia gravis. Nat. Rev. Dis. Primers 5:30. doi: 10.1038/s41572-019-0079-y, 31048702

[ref5] Girmenia C. Barcellini W. Bianchi P. Di Bona E. Iori A. P. Notaro R. . (2023). Management of infection in PNH patients treated with eculizumab or other complement inhibitors: unmet clinical needs. Blood Rev. 58:101013. doi: 10.1016/j.blre.2022.101013, 36117056

[ref6] Howard J. F. Bresch S. Farmakidis C. Freimer M. Genge A. Hewamadduma C. . (2024). Long-term safety and efficacy of Zilucoplan in patients with generalized myasthenia gravis: interim analysis of the RAISE-XT open-label extension study. Ther. Adv. Neurol. Disord. 17:17562864241243186. doi: 10.1177/17562864241243186, 38638673 PMC11025429

[ref7] Howard J. F. Bresch S. Genge A. Hewamadduma C. Hinton J. Hussain Y. . (2023). Safety and efficacy of Zilucoplan in patients with generalised myasthenia gravis (RAISE): a randomised, double-blind, placebo-controlled, phase 3 study. Lancet Neurol. 22, 395–406. doi: 10.1016/S1474-4422(23)00080-7, 37059508

[ref8] Huan X. Chen R. Jin L. Wang Y. Xu Y. Yang L. . (2026). 1-year clinical outcome post-myasthenic crisis: a Multicenter prospective study in China. Eur. J. Neurol. 33:e70513. doi: 10.1111/ene.70513, 41589418 PMC12836041

[ref9] Ito S. Sugimoto T. Naito H. Aoki S. Nakamori M. Miyachi T. . (2025). Zilucoplan for successful early weaning from mechanical ventilation and avoiding tracheostomy in an 85-year-old woman experiencing myasthenic crisis: a case report. Cureus 17:e80203. doi: 10.7759/cureus.80203 40190980 PMC11972557

[ref10] Maruani A. Demeret S. Piljan M. Weiss N. Marois C. (2025). Zilucoplan in myasthenic crisis refractory to conventional treatment: a report of two cases. Acta Neurol. Belg. 125, 1689–1691. doi: 10.1007/s13760-025-02860-6, 40892124

[ref11] Roper J. Fleming M. E. Long B. Koyfman A. (2017). Myasthenia gravis and crisis: evaluation and management in the emergency department. J. Emerg. Med. 53, 843–853. doi: 10.1016/j.jemermed.2017.06.009, 28916122

[ref12] San P. P. Jacob S. (2023). Role of complement in myasthenia gravis. Front. Neurol. 14:1277596. doi: 10.3389/fneur.2023.1277596, 37869140 PMC10585143

[ref13] Sanders D. B. Wolfe G. I. Benatar M. Evoli A. Gilhus N. E. Illa I. . (2016). International consensus guidance for management of myasthenia gravis: executive summary. Neurology 87, 419–425. doi: 10.1212/WNL.0000000000002790, 27358333 PMC4977114

[ref14] Tang G. Q. Tang Y. Dhamnaskar K. Hoarty M. D. Vyasamneni R. Vadysirisack D. D. . (2023). Zilucoplan, a macrocyclic peptide inhibitor of human complement component 5, uses a dual mode of action to prevent terminal complement pathway activation. Front. Immunol. 14:1213920. doi: 10.3389/fimmu.2023.1213920, 37622108 PMC10446491

[ref15] Vinciguerra C. Bevilacqua L. Toriello A. Iovino A. Piscosquito G. Calicchio G. . (2023). Starting eculizumab as rescue therapy in refractory myasthenic crisis. Neurol. Sci. 44, 3707–3709. doi: 10.1007/s10072-023-06900-y, 37306795

